# Clinical and Genetic Spectrum of Large AIP Deletions

**DOI:** 10.1155/humu/4212742

**Published:** 2026-09-12

**Authors:** Tea Shehu Kolnikaj, Maren Ruka, Artur Xhumari, Anila Babameto-Laku, Athanasios Siolos, Panagiotis Alexandros Drakos, Kesson Magid, Thomas O. Millner, Robert D. Murray, Marinella Tzanela, Dragana Miljić, Arlinda Ramaj, Paul Benjamin Loughrey, Stelios Tigas, Márta Korbonits

**Affiliations:** ^1^ Endocrinology, American Hospital, University of Medicine, Tirana, Albania, amerikanhastanesi.org; ^2^ Department of Clinical and Molecular Medicine, Sapienza University of Rome, Rome, Italy, uniroma1.it; ^3^ Neurosurgery, Faculty of Medicine, University of Medicine, Tirana, Albania, unito.it; ^4^ Genetics, Faculty of Medicine, University of Medicine Tirana, Tirana, Albania; ^5^ Department of Endocrinology, Ioannina University Hospital, Ioannina, Greece, uoi.gr; ^6^ Endocrinology, William Harvey Research Institute, Barts and the London School of Medicine, Queen Mary University of London, London, UK, qmul.ac.uk; ^7^ Neuropathology, Blizard Institute, Barts and the London School of Medicine, Queen Mary University of London, London, UK, qmul.ac.uk; ^8^ Endocrinology, University of Leeds, Leeds, UK, leeds.ac.uk; ^9^ Endocrinology, Evangelismos Hospital, Athens, Greece, evaggelismos-hosp.gr; ^10^ Clinic for Endocrinology, Diabetes and Metabolic Disorders, University Clinical Centre of Serbia, Medical Faculty, Belgrade University, Belgrade, Serbia, bg.ac.rs; ^11^ Local Health Care Unit, Department of Epidemiology and Environmental Health, Tirana, Albania; ^12^ Department of Endocrinology, Beaumont Hospital, Dublin, Ireland, beaumont.edu

**Keywords:** acromegaly, AIP gene, familial isolated pituitary adenoma, genetic mutation, Hürthle cell thyroid carcinoma

## Abstract

Familial isolated pituitary adenoma (FIPA) accounts for approximately 2%–5% of all pituitary adenomas, with inactivating variants of the aryl hydrocarbon receptor‐interacting protein (*AIP*) gene representing the most frequent known genetic cause. Clinically, patients with *AIP* variants often have young‐onset macroadenomas with growth hormone hypersecretion, although disease severity and penetrance are variable. Most reported *AIP* variants are point mutations, whereas large deletions are rare and potentially underdiagnosed. Accurate detection of *AIP* copy‐number variants requires methods such as multiplex ligation‐dependent probe amplification or validated copy‐number analysis of next‐generation sequencing data, as Sanger sequencing alone may fail to identify these alterations. Due to the rarity of the disease, it is unknown whether large deletions in the ubiquitously expressed *AIP* gene are associated with potentially more severe phenotype. Available data suggest that large deletions may occur in 8%–10% of *AIP* mutation‐positive pedigrees, highlighting the importance of incorporating copy‐number variant detection into *AIP* testing workflows. We analysed data from all published patients with large *AIP* deletions (*n* = 25) and report here two novel large *AIP* deletions (Exons 3–4 and Exons 2–6 deletions) and three additional three families, including an Albanian kindred associated with metastatic Hürthle cell thyroid carcinoma. No major differences compared with other *AIP* variants were found in age at diagnosis, tumour size, hormonal profile, sex distribution or presence of other tumours. A role for *AIP* variants in thyroid carcinogenesis is unlikely.

## 1. Introduction

Pathogenic variants in the *AIP* gene are the most frequent genetic cause of familial isolated pituitary adenoma (FIPA), a clinical entity that accounts for a small percentage of pituitary adenomas/pituitary neuroendocrine tumours [1]. FIPA is defined by the occurrence of pituitary adenomas in at least two family members, in the absence of other syndromic features or in an individual with a germline pathogenic variant [2]. Since their first description in 2006 [3], *AIP* variants have been identified in 10% of FIPA families, with clinical penetrance estimated at 17%–23% [1, 4–7]. The predominant clinical phenotype is a growth hormone (GH)‐secreting adenoma—up to 80% of cases—followed by prolactinomas and, less frequently, clinically nonfunctioning pituitary tumours. If these latter ones are operated, GH, prolactin or PIT1 staining is usually positive [5, 8]. A few cases of pituitary hyperplasia with GH excess have been described [9, 10], whereas most germline *AIP* variants reported to date are point mutations or small insertions/deletions, large deletions—representing a type of copy‐number variant (CNV)—span one or more exons of the *AIP* gene. They have been rarely identified and probably underrecognized [11]. Their detection usually requires multiplex ligation–dependent probe amplification (MLPA) or validated copy‐number analysis from next‐generation sequencing data, as Sanger sequencing is not an appropriate method for detecting CNVs, such as large deletions [11].

*AIP*‐related pituitary disease exhibits a wide spectrum, ranging from (i) patients with young‐onset aggressively growing pituitary tumours [1, 3, 10, 12–14], (ii) hyperplasia, slow or indolent disease courses [9, 10, 15], (iii) patients with pituitary lesions not dissimilar to incidentalomas, usually identified through prospective clinical assessment of carriers [5, 16], as well as (iv) the majority of carriers, unaffected throughout life [3, 6]. The clinical impact of *AIP* mutations varies significantly [17] and there is no explanation for the variability of penetrance and expressivity, as a clear genotype–phenotype correlation across *AIP* mutations has not been demonstrated [5].

In this review, we summarize the current landscape of large *AIP* deletions, with emphasis on their epidemiology, detection methodologies and clinical implications. We report here two novel large deletions and three novel kindreds with familial acromegaly or gigantism due to large deletions. As an illustrative example of some of the complexities, we describe in detail a family with acromegaly due to a large *AIP* deletion and the coexistence of Hürthle cell thyroid carcinoma (Boxes 1 and 2).

**Figure 1:**
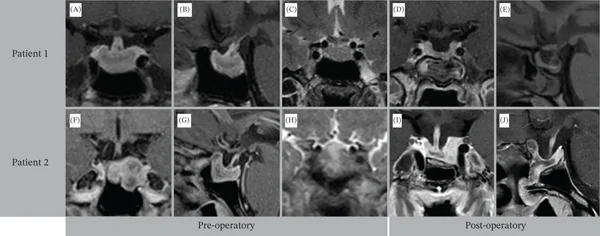
Preoperative and postoperative MRI scans of (A–E) Patient 1 and (F–J) Patient 2. Patient 1: Preoperative MRI (T1‐weighted, contrast‐enhanced: (A) coronal; (B) sagittal) shows a 16 × 11 *m* *m* heterogeneous sellar mass without suprasellar extension, with slight stalk thickening but no deviation. The (C) coronal T2‐weighted image demonstrates a mildly T2‐hyperintense pituitary macroadenoma. Postoperative MRI obtained 2 years after surgery (T1‐weighted, contrast‐enhanced: (D) coronal; (E) sagittal) reveals a partially empty sella with normal pituitary signal intensity and no residual tumour. Patient 2: Preoperative scans (T1‐weighted, contrast‐enhanced: (F) coronal; (G) sagittal) show a 23 × 15 × 10 *m* *m* sellar mass extending into the left cavernous sinus without optic‐chiasm compression. (H) Coronal T2‐weighted image shows a hyperintense pituitary mass. Postoperative MRI (T1‐weighted, contrast‐enhanced: (I) coronal; (J) sagittal) demonstrates residual enhancing elements in the left cavernous sinus.

**Figure 2:**
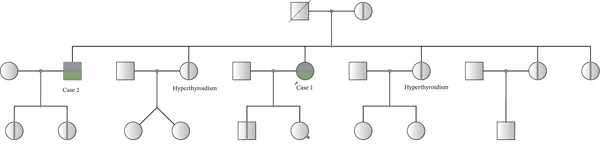
Family pedigree. The family pedigree illustrates two patients with acromegaly (grey colour) and *AIP* mutation (green colour). There are nine first‐degree relatives with 50% chance to be carriers. Patient 1′s daughter (asterisk) is not carrying the mutation. The other first‐degree family members (vertical line) were invited for genetic testing. Two sisters have hyperthyroidism.

**Box 1: Patient 1.**
A 39‐year‐old woman presented with a long‐standing history of acromegalic features, which began at the age 20, shortly after the delivery of her first child. She reported progressive enlargement of her hands and feet, necessitating a change in her shoe size (from 38 to 39 EU) and a need to change her wedding ring size. She noted changes in her facial bone structure, lips, nose and tongue. Additionally, she experienced oily skin, excessive sweating and a deepening of her voice. The patient also described severe headaches without aura, localized to the temporal, frontal and parietal regions. Following the birth of her son at the age of 20, she had amenorrhea for approximately 4 years and struggled with infertility in the following 12 years, despite seeking medical assistance and undergoing multiple tests (details unavailable). At the age of 33, she conceived naturally and delivered a healthy girl. At age 37, she was diagnosed with diabetes mellitus and was started on metformin 500 mg twice daily. The following year during a routine consultation for diabetes, her acromegalic features were noted by her physician, prompting a referral for further investigation. The patient′s growth and pubertal development were reported as normal (menarche at 13 years). She was 165 cm (midparental height 158 cm). IGF‐1 was 1033 ng/mL, 3.3x upper limit of normal, random GH was 30.1 ng/mL. Prolactin levels were 33.4 ng/mL (normal range 1.9–25 ng/mL). The rest of the pituitary function was normal. Pituitary MRI revealed a 16 × 11 mm homogenous sellar mass with no evidence of suprasellar extension or cavernous sinus invasion with slight stalk thickening without deviation (Figure 1A–C). Transsphenoidal surgery was performed, and histology identified a sparsely granulated somatolactotroph tumour with widespread fibrous bodies; Ki67 could not be assessed due to low quality tissue preservation. Following surgery, her menstrual cycle normalized within 2–3 months, and no anterior pituitary deficiencies were detected. Follow‐up MRI examinations 2 (Figure 1D,E) and 5 years after surgery showed no recurrence with a partially empty fossa. At her last follow‐up, age 51 years, 12 years after her diagnosis and surgery, the patient reported no symptoms and had normal visual fields on formal testing. She has an asymptomatic multinodular goitre on ultrasound and cardiac ultrasound showed a normal‐sized left ventricle with preserved ejection fraction, no wall motion abnormalities, normal ascending aorta, left atrium and there was no pericardial effusion. Her biochemical assessment showed slightly above random GH (2 ng/mL) and normal IGF‐1 levels (164 ng/mL; (age‐adjusted normal range 48.1–209 ng/mL). Genetic testing for *AIP* mutations (next generation sequencing) identified a pathogenic large deletion of Exons 2 and 3. Thyroid and cortisol axes were normal, LH and FSH were in perimenopausal ranges. She is still under treatment for diabetes. Her older brother (Patient 2) was diagnosed with acromegaly 8 years after her diagnosis (Box 2). She has 2 sisters with hyperthyroidism. The family pedigree is seen on Figure 2.

**Box 2: Patient 2.**
A 42‐year‐old man presented with progressive enlargement of his hands and feet, coarse facial features, keloid acne, macroglossia, fatigue and a recent diagnosis of Type 2 diabetes mellitus. His symptoms started approximately 10 years earlier. He reported having one sister with acromegaly diagnosed at the age of 34 (Patient 1) (Figure 2). His height was 178 cm, midparental height of 171 cm. In addition to the acromegalic features, physical examination revealed a nodular goitre. Serum IGF‐1 was 676 ng/mL (110–215) and GH > 35 ng/mL (0.01–0.97), with a lack of GH suppression during OGTT (nadir GH > 34 ng/mL). There was no hyperprolactinaemia and the rest of pituitary function was normal. There was no clinical or laboratory evidence of MEN syndrome or Carney complex (normal serum calcium and phosphate levels, no evidence of cardiac myxomas on echocardiography, normal abdominal CT, no skin lesions). MRI revealed a 23 × 15 × 10 mm pituitary macroadenoma extending into the left cavernous sinus without optic chiasm compression (Figure 1F–J). Following the diagnosis of acromegaly, he was referred for transsphenoidal surgery and he was started on octreotide LAR 20 mg and then increased 30 mg monthly. In the meanwhile, however, fine needle aspiration of a 2 cm nodule in the left thyroid lobe showed Bethesda Category VI, and the patient underwent total thyroidectomy. Histology was consistent with oncocytic (Hürthle cell) thyroid carcinoma with capsular and vascular invasion (pT3NXM0 at initial staging). Radioactive iodine was administered 3 months later (127 mCi). He underwent transsphenoidal resection of the pituitary adenoma 8 months later. The histology confirmed a sparsely granulated somatolactotroph tumour with fibrous bodies; Ki67 could not be assessed reliably due to poor preservation of the tissue. Postoperatively there was evidence of persistent disease (IGF‐1 414 ng/mL [110–215] and random GH: 29 ng/mL). Gadolinium‐enhanced MRI 2 years later showed left cavernous sinus residua (Figure 1I). Following treatment with monthly octreotide‐LAR 30 mg, IGF‐1 levels were reduced into the normal range (145 ng/mL) and random GH was 4.57 ng/mL. MRI 40 months after the surgery showed < 20% shrinkage of the tumour. The rest of the pituitary function was normal. Genetic testing for AIP mutations (next generation sequencing) identified a pathogenic large deletion of Exons 3 and 4; the same variant was identified in his sister. Considering the thyroid cancer, the patient received several courses of radioactive iodine therapy due to persistent localized disease (thyroid bed, cervical lymph nodes) up to a cumulative dose of 577 mCi. He did not wish to have further surgical treatment. Three years after thyroidectomy there was evidence of RAI refractory lung metastases. Next generation sequencing of thyroid tumour‐derived DNA showed pathogenic single nucleotide variants of the MSH6, TP53 and SMARCA4 genes and deletion of the HLA‐B gene. No loss‐of‐heterozygosity was observed at the Chr11.q13 locus. He is currently treated with lenvatinib. Cardiac MRI showed hypertrophic cardiomyopathy with preserved ejection fraction, increased left ventricular absolute mass, asymmetric myocardial thickening, late gadolinium enhancement of the left ventricle and interventricular septum. Stage II hypertension was also diagnosed and treated with amlodipine 5 mg.

## 2. Methods and Results

A literature search was performed in PubMed and Google Scholar to identify patients reported with pituitary adenoma due to large *AIP* deletions using the keywords ‘AIP’, ‘large deletion’, ‘FIPA’, ‘familial isolated pituitary adenoma’, ‘pituitary adenoma’, ‘PitNET’, ‘pituitary neuroendocrine tumour/tumor’. Additional articles were identified through manual screening and searches in other databases including ClinVar, Decipher and Cosmic. Seven studies reported patients with large *AIP* deletions.

In this manuscript, we report three new families (Kindreds 1, 8 and 9) with two novel large deletions (Exon 2–6 deletion and Exons 3–4 deletion, Table 1, Boxes 1 and 2). We have expanded our own previous data [5] with further family members of the previously published Kindreds 3, 11 and 12 (Table 1).

**Table 1:** Clinical and genetic features of kindreds with large *AIP* deletions.

Family ID and reference	Country of origin	Clinical diagnosis	Immunostaining of pituitary tumour	Sex	Age at diagnosis (year)	Size of pituitary tumour	*AIP* variant	Comments and data for other family members
*Kindred 1 (current manuscript)*	*United States*	*Gigantism*	*GH/PRL*	*M*	*19*	*Macroadenoma*	*Exon 1 deletion*	*Two carriers, normal findings*
		*Gigantism*	*GH*	*M*	*17*	*Macroadenoma*		

*Kindred 2* [5]	*United Kingdom*	*Acromegaly*	*GH*	*F*	*19*	*Macroadenoma*	*Exon 1 deletion*	*Sporadic*

*Kindred* 3 [11, 16]	*United Kingdom*	*Pituitary apoplexy*	*GH/PRL*	*M*	*18*	*Macroadenoma*	*Exon 2 deletion*	^∗^ **At the age of 17 she had a 4**‐mm **adenoma, which by the age of 34 was reduced to 2** mm. One carrier (died at the age of 41) ependymoma. *Nine carriers: normal findings*
		*Gigantism*	*GH/PRL, also 20% LH/FSH positive cells*	*M*	*18*	*Macroadenoma*		
		*Acromegaly and prolactinoma*	*GH and PRL in 2 separate lesions*	*F*	*26*	*Two microadenomas*		
		*NFPA°*	*Not operated*	*F*	*17*	*Microadenoma/Rathke's cyst* ^∗^		
		*NFPA°*	*Not operated*	*F*	*17*	*Microadenoma*		

*Kindred* 4 [15]	*Brazil*	*Gigantism*	*GH*	*M*	*19*	*Giant adenoma*	*Exon 2 deletion*	*Sporadic*

*Kindred* 5 [11, 18]	*Germany*	*Acromegaly*	*GH*	*F*	*46*	*Macroadenoma*	*Exons 1–2 deletion*	*Data not available*
		*Acromegaly*	*GH/PRL*	*M*	*24*	*Macroadenoma*		

*Kindred* 6 [19]	*Germany*	*Gigantism*	*NA*	*M*	*19*	*Invasive Macroadenoma*	*Exons 1–2 deletion*	*Data not available*

*Kindred 7* [19]	*Germany*	*Acromegaly*	*NA*	*F*	*19*	*Invasive Macroadenoma*	*Exons 1–2 deletion*	*Sporadic*

*Kindred 8 (current manuscript)*	*United States*	*Acromegaly*	*NA*	*F*	*27*	*Microadenoma*	*Exons 2–6 deletion*	*One obligate carrier, no known endocrine disease*
		*Gigantism*	*NA*	*M*	*14*	*Macroadenoma*		

*Kindred 9 (current manuscript)*	*Albania*	*Acromegaly*	*GH/PRL*	*F*	*39*	*Macroadenoma*	*Exons 3–4 deletion*	*No carrier family members have been identified (yet). Metastatic Hürthle cell carcinoma in male patient*
		*Acromegaly*	*GH/PRL*	*M*	*42*	*Macroadenoma*		

*Kindred 10* [20]	*United Kingdom*	*Gigantism*	*GH*	*M*	*35*	*Macroadenoma*	*Exons 1–6 deletion*	*Four carriers, none known to be affected*
		*Gigantism*	*GH*	*M*	*19*	*Macroadenoma*		
		*Gigantism*	*GH*	*M*	*23*	*Macroadenoma*		
		*Acromegaly°*	*GH*	*M*	*38*	*Macroadenoma*		

*Kindred 11* [20]	*Serbia*	*Gigantism*	*GH*	*M*	*20*	*Macroadenoma*	*Exons 1–6 deletion*	*Carrier parent no obvious pituitary disease (died of other reasons). Benign* 30 × 25 × 20 *m* *m* *nonfunctioning adrenocortical adenoma was operated in the male patient with gigantism at age 40* years old
		*Gigantism*	*GH*	*F*	*10*	*Macroadenoma*		

*Kindred 12* [15]	*Greece*	*Acromegaly*	*Not operated*	*M*	*32*	*Giant adenoma*	*Exons 1–6 deletion*	*One carrier clinically well*
		*Prolactinoma*	*Not operated*	*F*	*26*	*Microadenoma*		

*Note:* Patients gave written consent to report their data. The symbol ‘°’ indicates prospectively diagnosed case. The asterisk identifies the patient corresponding to the clinical details provided in the accompanying notes; the bold emphasis is used to facilitate identification.

Abbreviation: PRL, prolactin.

Regarding frequency of large deletions families: our cohort consists of 129 *AIP* mutation positive kindreds. Taking into account that our cohort harbours two 25–30 century‐old founder mutations [12, 21], the nine independent kindreds with large *AIP* deletions represent 10% of the independent kindreds with pathogenic/likely pathogenic *AIP* variants. The frequency of large *AIP* deletions was studied in different ways in the literature. One study examined FIPA families—including index patients with GH‐ (*n* = 10) or prolactin‐secreting (*n* = 4) tumours or with clinically nonfunctioning tumours (*n* = 7)—who were negative on Sanger sequencing for *AIP* mutations. They identified 2/21 kindreds (~10%) with large *AIP* deletion [11] (Table 1, Kindred 3 and 5). Another study examined 91 < 30‐year‐old patients with GH excess and found four patients with *AIP* mutation; two of these—apparently independent—kindreds harboured the same large deletion [19] (Table 1, Kindred 6 and 7) and this large deletion was previously described from the same country (Table 1, Kindred 5 [11]), raising the possibility of a common ancestor.

Combining our novel data and data from the literature, there are 25 patients from 12 kindreds with large *AIP* deletions (Table 1). There are eight kindreds with more than one affected subject (21 patients) and four simplex cases. Three of the patients included were prospectively diagnosed following genetic and clinical screening of family members (Table 1). The median age at diagnosis for affected patients was 19 years (range 10–46). There was male predominance (15 males, 10 females). Of the 25 patients identified, 18 patients had macroadenomas, two had giant adenomas. Clinical GH excess was present in 21 patients (84%). Eleven patients presented with gigantism (defined according to Hernández‐Ramírez et al. [15]) with GH (*n* = 7), GH/prolactin (*n* = 1) and GH/prolactin and 20% LH/FSH‐positive cells (*n* = 1) on immunostaining, whereas for two patients with gigantism immunostaining was not available. Out of the 10 patients with acromegaly, four had positive GH staining, three had GH/prolactin staining, one was not operated and for two no immunostaining data are available. Two cases had prolactinomas: one patient with microprolactinoma (not operated) and another had two separate microadenomas (a somatotroph and a lactotroph lesion [16]). Three patients had clinically nonfunctioning pituitary lesions. Two were diagnosed prospectively; one of these seemed to be a Rathke′s cleft cyst or a microadenoma on serial MRIs, showing a gradual decrease in size from 4 to 2 mm during follow‐up and another patient presented with pituitary apoplexy and had no endocrine evaluation before surgery; histopathology showed positive GH‐ and prolactin‐staining, although the patient did not exhibit clear clinical features of either acromegaly or prolactinoma.

Considering other tumour types in patients with large *AIP* deletions, a benign adrenal adenoma was found 18 years after the diagnosis of GH excess (Kindred 11) and an ependymoma was found in a carrier patient (not affected by pituitary disease) who died of that disease (Kindred 3). We report here an oncocytic (Hürthle cell) thyroid carcinoma in Kindred 9, where no loss‐of‐heterozygosity was present at the Chr11.q13 locus in the thyroid tumour (Box 2). No other patients harbouring a large *AIP* deletion have been described with a thyroid carcinoma [2, 5, 20].

Statistical comparison of clinical and tumour characteristics between patients with large *AIP* deletions and those reported in two large cohorts (excluding cases with large deletions) [1, 5] is presented in Table 2. No statistically significant differences were found across cohorts (*p* > 0.05) for age of diagnosis, sex, presence of gigantism in GH excess patients and tumour type. Although a statistically significant difference was observed in the proportion of macroadenomas between the Daly cohort and patients in the large deletion group (*p* = 0.048), this finding should be interpreted with caution given the differences in patient inclusion criteria and small sample size. There was no significant difference in this parameter when compared with the Marques cohort. Furthermore, these findings suggest no evidence of genotype‐phenotype correlation between large *AIP* deletions and other *AIP* variants. Overall, patients with large *AIP* deletions predominantly present with GH‐secreting adenomas, showing similar demographic and tumour features to those with other *AIP* variants.

**Table 2:** Comparison of clinical characteristics between patients with large *AIP* deletions and previously published multicentre series (without large deletions).

Parameter	Large *AIP* deletion (25 patients)	Marques 2020 (153 patients without large deletions)^5^	*p*1 *value*	Daly 2010 (96 patients)^1^	*p*2 *value*
*M* *e* *a* *n* ± *S* *D*/median and range age of diagnosis (years)	Mean 24.12 ± 9.38; median 19 (range 10–46)	Mean 24.4 ± 12.2; median 21 (range 7–68) (*n* = 145)	0.90	Median 23 (range 8–74)	—
Male predominance (%)	60%	60.8% (*n* = 153)	1	63.5%	0.82
Macroadenomas (%)	80%	83.6% (*n* = 134)	0.77	93.3%	**0.048**
GH‐secreting (%)	84%	81.7% (*n* = 153)	1	78.1%	0.59
Gigantism	Within GH excess cohort 11/21 (52%)	Within GH excess cohort 67/125 (54%)	1	No data were reported to calculate this parameter	—

*Note:* *p*1 value is the comparison between the large‐deletion group and the Marques et al. cohort [5]. *p*2 value is the comparison between the large‐deletion group and the Daly et al. cohort [1]. Individual‐level (raw) data were available for the present cohort and were subsequently aggregated to enable comparison with the published datasets of Marques et al.^5^and Daly et al. [1]. Statistical analyses were performed in R (v4.4.0), applying Welch′s *t*‐test for continuous variables (age) and Fisher′s exact test for other categorical variables. *p* < 0.05 is considered statistically significant. The bolded value indicates the only statistically significant *p* value in the table.

## 3. Discussion

### 3.1. Clinical Features

Clinical features in patients with large *AIP* deletion are comparable with patients with other types of pathogenic/likely pathogenic *AIP* variants [1, 5]. The absence of a distinct phenotype implies that the underlying disease biology and clinical behaviour are driven more by loss of *AIP* function itself, rather than by the structural type of variant [5]. We compared tumour type (GH or prolactin), age of onset, tumour size and presence of gigantism between large deletion and other types of pathogenic/likely pathogenic *AIP* variants (Table 2), although due to the low number of large deletion cases reported, the assessment of these differences is not reliable. Although we have previously published on detailed assessment of AIP‐related disease penetrance [6], comparison of penetrance here could not be assessed due to the incomplete screening/data available for apparently unaffected family members.

In other pituitary tumour‐related genetic conditions, genotype–phenotype correlations are heterogeneous. *PRKAR1A*‐related Carney complex shows a clear genotype–phenotype correlation: Large deletions are associated with more severe phenotypes, including rare features like melanotic schwannoma in large deletion cases [22, 23]. For multiple endocrine neoplasia Type 1 (MEN1), as noted in the 2025 guidelines [24], no clear genotype–phenotype correlation is established [25]. However, some evidence suggests that truncating *MEN1* mutations (frameshift and nonsense variants) may be associated with a higher risk of aggressive phenotypes, such as gastro‐entero‐pancreatic neuroendocrine tumours, including pancreatic NETs. These associations, however, are primarily drawn from small cohorts or case reports [26]. Despite the identification of over 1336 mutations in the *MEN1* gene, no clear genotype–phenotype correlation has been found [27]. In MEN4, caused by *CDKN1B* mutations, the impact of large deletions on phenotype severity remains unclear, as most cases involve point mutations and data are still limited [28].

Male predominance in *AIP*‐mutations related tumours is incompletely understood. Part of this observation may reflect ascertainment bias: who is tested for *AIP* mutations. The later physiological onset of puberty in males compared with females may provide a longer prepubertal window for tumour growth before epiphyseal closure, increasing the likelihood of presenting with pituitary gigantism [15]. Women with acromegaly exhibit relatively lower IGF‐1 concentrations for a given GH level [29], related to oestrogen‐mediated suppression of hepatic IGF‐1 production. Those differences could theoretically attenuate the clinical expression of GH excess in females. According to animal studies, male GH cells generate more and larger clusters and have increased proliferative capacity compared with females [30, 31]. This could be another key predisposing factor for tumour development, if also present in humans.

### 3.2. Thyroid Cancer

The role of AIP in tumorigenesis is complex. Although it is clearly a tumour suppressor in the pituitary, it is upregulated in other cancers and is one of those controversial proteins that could be an oncogene or a tumour suppressor gene depending on the tissue context [32]. Current evidence suggests that lack of functional AIP does not contribute to extrapituitary tumorigenesis [15, 33–35].

Regarding thyroid tumours, in addition to the case presented here (Box 2), there is one other patient described with a pathogenic *AIP* mutation, GH excess and a thyroid carcinoma: an 18‐year‐old female with gigantism due to a p.Gln229∗ nonsense *AIP* variant, who had a tall‐cell variant of papillary thyroid carcinoma [36]. There are two cases of a follicular carcinoma described in patients with a pathogenic germline *AIP* variant (p.Gln285∗ and p.Leu115TrpfsTer41); however, these patients did not have GH excess [36, 37]. Our case has a Hürthle cell carcinoma, not previously described in patients with acromegaly and *AIP* mutations. In the COSMIC database (reporting on somatic variants in cancer samples, https://cancer.sanger.ac.uk/cosmic), there is a single case of recurrent Hürthle cell carcinoma with a somatic missense *AIP* variant c.593C>T; p.A198V (not present in gnomAD, in silico scores compatible with likely pathogenic variant: Revel score 0.877, Alpha‐missense score 0.9794). Although loss‐of‐heterozygosity was found in one of the follicular cancer samples at the *AIP* locus [36], follicular carcinomas are known to have loss of material at the 11q13 site, but typically the region repeatedly identified in follicular cancers—therefore thought to be the relevant region—is between markers D11S4946 (at chr11: 64811247, GRCh38/hg38) and D11S4939 (at chr11: 65000799), which is distant from the *AIP* gene region (chr11: 67,483,026–67,491,103) [38].

Thyroid abnormalities are common in patients living with acromegaly, with goitre prevalence ranging from 78%–92% by ultrasound and 11%–87% by palpation [39]. Differentiated thyroid cancers in patients with acromegaly are well documented [40, 41], occurring in up to 8%–11% of patients, underscoring the importance of awareness of potential thyroid disease in patients living with acromegaly [42, 43]. One possible mechanism involves IGF‐1, which promotes cell replication and inhibits apoptosis through IGF‐1 receptor signalling in thyroid follicular epithelial cells [44]. IGF‐1 may stimulate thyroid tumorigenesis by promoting proliferation via the MET/TP53/KLLN signalling pathway [45]. In line with this, the identification of pathogenic variants in *TP53* in our patient may be mechanistically relevant, supporting a potential link between IGF‐1 excess and disruption of p53‐dependent tumour suppressor pathways. However, guidelines do not support thyroid cancer screening in patients with acromegaly [42].

The rare subtype of thyroid carcinoma, oncocytic (Hürthle cell) carcinoma, characterized by eosinophilic and granular cytoplasm, accounts for only 3%–4% of all thyroid malignancies [46]. The coexistence of acromegaly and Hürthle cell carcinoma is a combination that, to our knowledge, has not been previously reported. Hürthle cell carcinoma is defined by a distinct interplay of nuclear and mitochondrial alterations that differentiate it from other thyroid malignancies. Nuclear mutations often involve the PI3K/AKT/mTOR and RAS/RAF/MAPK pathways (*PTEN, PIK3CA, TSC1/2, NRAS, HRAS*), whereas *TP53* and *TERT* promoter mutations associate with invasive, aggressive disease. Additional, but less frequent alterations include *EIF1AX, ARID1A, MEN1, DAXX* and *MED12*, indicating roles for chromatin and transcriptional dysregulation. Genomic instability is common, with chromosomal amplifications and widespread loss of heterozygosity. Mitochondrial DNA changes—particularly truncating mutations in Complex I subunits and large deletions—lead to oxidative phosphorylation failure and the oncocytic phenotype. Rare variants such as GRIM19 and TIMM44 also affect mitochondrial structure and function. Together, these nuclear and mitochondrial defects define a tumour driven by mitochondrial dysfunction, genomic instability and deregulated proliferative and metabolic signalling [46].

Next generation sequencing (NGS) of our patient′s Hürthle tumour‐derived DNA revealed pathogenic variants in *MSH6*, *TP53* and *SMARCA4*, together with a deletion of *HLA-B*. Amongst these, *TP53* mutations are well recognized in aggressive and dedifferentiated thyroid carcinomas, including Hürthle cell carcinoma [46]. Somatic *MSH6* alterations, which cause mismatch‐repair deficiency and microsatellite instability, are infrequently reported in thyroid cancer, whereas germline variants are usually linked to Lynch syndrome, a condition in which thyroid cancer is not part of the classical tumour spectrum [47, 48]. *SMARCA4* mutations have not been systematically reported in thyroid carcinoma, but functional studies show that its protein product BRG1 interacts with thyroid hormone receptor *β* to regulate chromatin accessibility and repress oncogenic transcription. Loss or inhibition of this BRG1‐mediated chromatin remodelling may therefore contribute to dedifferentiation and tumour progression [49]. The HLA‐B deletion, although not previously characterized in thyroid malignancy, aligns with immune‐escape mechanisms observed in other solid tumours [50]. Together, these findings expand the molecular spectrum of Hürthle cell carcinoma, highlighting potential contributions from DNA repair failure, chromatin dysregulation and immune evasion to its aggressive biological behaviour. The coexistence of this genetic profile with acromegaly in our patient appears most likely coincidental rather than mechanistically linked.

### 3.3. Genetic Testing—Germline

Germline genetic testing strategy for patients with pituitary tumours has now been included in some of the available guidelines [2, 24, 25, 42, 51–54]. Given the low frequency of germline mutations in apparently sporadic pituitary adenomas, routine genetic testing is not recommended for all patients. However, specific patient subgroups identified by phenotypic features would benefit from genetic testing. These include childhood‐onset disease, pituitary gigantism, macroadenomas, especially GH and prolactin‐secreting tumours before the age of 30 years and positive family history. Therefore, in addition to patients with potentially syndromic disease, for patients with these parameters genetic testing is recommended [52, 53, 55]. Current guideline for aggressive pituitary tumours applies similar indications [54]. The recent acromegaly guideline uses a younger age threshold (< 18 years) at presentation [42, 56]. However, data from our large cohort found that over half of *AIP* mutation‐positive patients without family history are older than 18 at diagnosis and 15% older than 30 years (Korbonits unpublished data). We believe that taking a careful history and considering age of symptom onset is a better parameter than age at diagnosis to decide if genetic testing is indicated or not, as using this parameter only a third of the *AIP* mutation‐positive patients reported first symptom over age 18 years and less than 10% above the age of 30 years ([5] and Korbonits unpublished data).

Most large genetic laboratories have now implemented NGS‐based multigene panels as an efficient approach to identify variants and more recently these NGS techniques are capable of picking up large deletions as well [57]. If Sanger sequencing is applied or exome sequencing with low capacity to pick up large deletions, then additional routine CGH array (targeting the whole genome), targeted CGH array (with high resolution in prespecified regions, capable of identifying smaller large deletions or region‐specific MLPA can be used to identify large deletions.

The policy on whether to apply single gene testing or multigene panels is variable. In some cases, single gene testing might be the most logical first step. For example, a childhood‐onset GH‐secreting macroadenoma with a sparsely granulated pattern on histology and positive family history of young‐onset acromegaly or prolactinoma and no personal or family history of syndromic disease (MEN1: hypercalcaemia, kidney stones or pancreas tumours, Carney complex: characteristic skin lesions, myxoma, testis tumours, adrenal hypercortisolism), then the chances of being positive for AIP are over 76% [58]. However, in most cases, a multigene panel approach is more suitable. In some countries, for example the United Kingdom, a small panel is for pituitary adenoma patients containing seven endocrine neoplasia genes (*AIP, CDC73, CDKN1B, MEN1, PRKAR1A*, *VHL* and *RET*), where the MAX gene, more recently established as a rare pituitary tumour gene, is not present. Laboratories in other countries are using large cancer panels, containing hundreds of cancer‐related genes (for example in the United States and others). In the new MEN1 guidelines, single‐gene testing is recommended in classic clinical presentations [24, 25]. There are clear advantages and disadvantages of both approaches. The balance between missing/identifying all possible variants as opposed to identifying variants not corresponding to the phenotype is a perplexing question, which needs to be further discussed by the clinical genetic community.

### 3.4. Genetic Testing—Somatic

Loss‐of‐function *AIP* mutations in the pituitary follow the classical pattern of the Knudson hypothesis of tumour suppressor genes, where a germline mutation in combination with a somatic ‘second hit’ leads to complete AIP deficiency in the tumour [32]. Losing a functional second copy could have different mechanisms; the most common one is a large deletion affecting the healthy (i.e., nonmutated) copy of chromosome 11. This method was used to narrow down the location of the gene before it was actually identified [59]. Loss‐of‐heterozygosity has been demonstrated in numerous studies for pathogenic/likely pathogenic missense and nonsense variants, whereas the lack of loss‐of‐heterozygosity was seen in variants unlikely to be pathogenic [13, 33, 60, 61]. Somatic testing for a “second‐hit” is not routinely performed, but can be considered in cases where the pathogenicity of the germline variant is questionable and the presence of loss‐of‐heterozygosity would add to the assessment of the variant [60]. *AIP* mutations as disease‐causing somatic variants, without an accompanying germline variant, have been seen extremely rarely [10]. AIP functions as a cochaperone protein in the pituitary and its loss predisposes mainly to PIT1‐lineage pituitary tumours such as somatotroph and lactotroph adenomas through different mechanisms [1, 2, 4, 5]. *AIP* mutations disrupt key cellular processes—including cAMP/PKA signalling, RET‐mediated apoptosis and chromatin and receptor interactions—leading to enhanced proliferation and apoptosis resistance in PIT1 lineage cells [62–71]. A proinvasive tumour microenvironment characterized by macrophage infiltration, upregulation of tumour‐derived cytokines (CCL5) and epithelial‐mesenchymal transition‐pathway changes [72] with additional environmental modulation of AHR–AIP signalling [64], along with epigenetic repression by miR‐34 [70, 71], may further contribute to tumour aggressiveness and reduced responsiveness to somatostatin analogues.

## 4. Limitations

This study has several limitations. Genetic material from patients and family members from the literature was not available, so haplotype analysis could not be performed and it was not possible to assess founder effect. In addition, the small sample size due to the rarity of large *AIP* deletions limits strong conclusions regarding genotype‐phenotype correlation. We were also unable to assess the penetrance of the large deletions as not all family members were tested. Furthermore, comparisons with previously published cohorts were, in some cases, based on aggregated data rather than individual‐level data, limiting the precision and consistency of cross‐study statistical analyses.

## 5. Conclusion

Our data suggest that kindreds with large *AIP* deletions do not have a different phenotype compared with kindreds with other types of pathogenic *AIP* variants. These cases underscore the importance of recognizing familial cases and genetic testing with methods that can detect large deletions in order to provide the possibility of early diagnosis in young family members.

## Author Contributions

T.S.K. recruited patients, collected and analysed the data, performed the literature review and drafted the manuscript. M.R. and A.X. contributed with neurosurgical expertise, clinical data acquisition and manuscript revision. A.B‐L. and K.M. performed the genetic analyses and contributed to manuscript revision. A.S., R.D.M., M.T., D.M. and S.T. contributed to patient recruitment, sample collection and interpretation of clinical data. P.A.D., A.R. and P.B.L. contributed to data analysis and manuscript revision. T.O.M. provided histopathological expertise and contributed to manuscript revision. M.K. conceived and supervised the study, contributed to patient recruitment and genetic and clinical data analysis, supervised the preparation of the manuscript and critically revised the manuscript.

## Funding

This study was supported by the Barts Charity (10.13039/100015652) and European Society of Endocrinology (10.13039/100010382).

## Disclosure

All authors approved the final version of the manuscript and agreed on the contribution statement.

## Ethics Statement

This manuscript reports several novel families and is also a review of the published literature. Reported patients were deidentified prior to analysis and publication and provided written informed consent for genetic testing and for the publication of anonymized genetic and clinical data. The study was conducted in accordance with the principles of the Declaration of Helsinki.

## Conflicts of Interest

M.R. received lecture fees from Recordati and attended advisory boards for Recordati and Camurus. P.B.L. attended an advisory board for Camurus. M.K. received lecture fees from Ipsen and Sandoz, consulted Corcept, Camurus, ONO and had grant support from NovoNordisk.

## Data Availability

The data that support the findings of this study are available from the corresponding author upon reasonable request.
